# The Late Agitation Arising from an Advertisement

**Published:** 1878-10

**Authors:** 


### THE LATE AGITATION ARISING FROM AN ADVERTISEMENT.

Members of the profession in Chicago have lately been very justly excited by the appearance in the daily papers of an advertisement which contained the names of a number of leading physicians of the city. To the names of these gentlemen were appended the professional and other degrees to which they were entitled, such as "A. M.," " M. D.," "Professor of———in——— Medical College," etc. The advertisement in question was written with so much skill as to make it appear that a large number of the professors in the medical colleges of the city, had ornamented their own names with the honorary appendages which they happen to possess. In order to emphasize the perfection of the articles advertised and recommended by the distinguished gentlemen who appeared to be his patrons, the author of the advertisement had it placed on the first page of the most extensively circulated daily journals, with a wood-cut at the top, so prominent as to preclude the possibility of its being overlooked by the most careless observer. This figure and long array of professors' names, made it the most conspicuous and attractive advertisement in the paper, and it doubtless served its purpose admirably. Immediately upon its appearance, and indeed very naturally, there was a free expression of surprise and indignation in medical circles. It was surprising that men of acknowledged merit and recognized position should lend their names to such a cause, and should in such ostentatious manner parade their titles before the public in connection with a subject that had no relation to the advancement of the welfare of the profession. The younger men were very properly indignant that their trusted seniors had violated the code of propriety, in so flagrant a manner.

The Chicago Medical Society took the initiative in passing resolutions requesting the erring ones to explain their position in the question at issue. Some responded to these resolutions, giving such explanation as seemed to them suitable to the occasion; others, however, did not think it necessary or proper to make any such response. The society after much warm discussion, exonerated from intentional wrong some of those who had offered explanation, and afterward indefinitely postponed consideration of the entire subject. No specific charges were made against any one, consequently no effort was made to administer discipline.

It is right that the profession should have a explanation of this disagreeable affair, that will enable those who desire it to avoid such unseemly and offensive publicity in future, and that will offer to those who feel most aggrieved at least an excuse for the apparently inexcusable facts. An optician who had recently established himself in Chicago, determined to convince some of its leading citizens that his work was greatly superior to anything heretofore offered to their inspection. Whatever the main fact may be, he succeeded by a painstaking exhibition of his wares and apparatus for making and adjusting his glasses, together with certificates of approval from many eminent politicians, lawyers, and physicians throughout the country, in making many of those who called upon him believe that he understood the subject upon which he was talking, and was an enthusiast in his calling. When he thought he had succeeded in proving the superiority of his wares to the satisfaction of his hearers, he would very politely request them to say so in writing. Those who complied with this request signed a note to that effect by appending their names. We believe in no instance was any degree or professional title found afterward attached to the names of such signers, nor was any verbal or written authority given for their subsequent use. We do not believe that any of the professional gentlemen concerned, knew to what use these signatures were to be put. We are furthermore certain that some of the signers at least, would never have consented to such ostentatious parade before the public. The whole thing was managed most skillfully. There was no formal certificate to which the signatures were attached. The formidable and captivating array of names, degrees and titles was simply headed, "recommended by."

When the attention of the signers was called to the subject, some were willing to justify themselves by the reflection that the act was not in violation of medical ethics, and that it was nothing more nor less than a recommendation to the public of the work of an accomplished and enterprising mechanic, while others felt unable to justify themselves upon any other grounds than that they had been imposed upon by the advertiser, who had presumed to do in their names all that was unseemly and offensive to professional propriety.

Such instances of involuntary participation in offensive advertisements have, however, been of too frequent occurrence lately to be easily excused, and, as there is no redress after the matter has once received the finishing touch of an artistic manipulator, the profession has a right to demand that its members see to it in future that they be not outwitted by men in search of influential names with which to forward their individual interests. The impression produced by this unfortunate occurrence will not be lost on members of the profession here, who not only desire to be right, but who wish to avoid all appearance of wrong; and we venture to hope, indeed we confidently believe, that they will never be outraged in this way again.

Upon another page of this number will be found communications from two of the gentlemen to whom reference was made by a correspondent in our last issue. In a spirit of impartiality we have thus given space to representatives of each side of the question at issue, for an expression of opinion. No further discussion of the subject will be conducted in these columns, as we consider that whatever good might have been the result of such discussion, has been already accomplished.

